# The cumulative dose-dependent effects of metformin on the development of tuberculosis in patients newly diagnosed with type 2 diabetes mellitus

**DOI:** 10.1186/s12890-021-01667-4

**Published:** 2021-09-25

**Authors:** Eunyoung Heo, Eunyoung Kim, Eun Jin Jang, Chang-Hoon Lee

**Affiliations:** 1 Department of Internal Medicine, SNU-SMG Boramae Medical Center, Seoul, Republic of Korea; 2 Department of Statistics, Kyungpook National University, Daegu, Republic of Korea; 3 Department of Information Statistics, Andong National University, Andong, Republic of Korea; 4 Division of Pulmonary and Critical Care Medicine, Department of Internal Medicine, Seoul National University Hospital, Seoul, Republic of Korea

**Keywords:** Diabetes mellitus, Tuberculosis, Metformin, Prevention, Effect

## Abstract

**Background:**

Diabetes mellitus (DM) is a well-known risk factor for tuberculosis (TB). Metformin, which is an essential anti-diabetic drug, has been shown to exhibit anti-TB effects in patients with DM. Its effect on preventing the development of TB among patients who are newly diagnosed with DM remains unclear.

**Methods:**

This was a retrospective cohort study using the claims database of the Korean Health Insurance Review and Assessment Service. The study population included patients who were newly diagnosed with type 2 DM and who were treated with anti-diabetic drugs between 1 January 2003 and 31 March 2011. A patient was defined as a metformin user if he/she had taken metformin for more than 28 days within 6 months since cohort entry, and as a metformin non-user if he/she had never been treated with metformin. The development of TB within 2 years after the index date was compared by Cox proportional hazard regression models between metformin users and 1:1 propensity score (PS)-matched non-users.

**Results:**

Among 76,973 patients who were newly diagnosed with type 2 DM, 13,396 were classified as metformin users, 52,736 were classified as metformin non-users, and 10,841 were excluded from the final analysis. PS-matched Cox proportional hazard regression models revealed that metformin use was not associated overall with the prevention of TB development (HR 1.17; 95% CI 0.75–1.83; *P* = 0.482). There was a trend, however, towards a reduction in the development of TB among patients taking a higher cumulative dose of metformin. Patients who were in the highest quartile (Q4) of cumulative metformin dose had only a 10% risk of developing TB compared to metformin non-users. In contrast, during the early phases of metformin treatment, patients in the second quartile (Q2) of cumulative metformin use had a higher risk of developing TB than patients in the first quartile (Q1).

**Conclusions:**

Only the highest cumulative doses of metformin were protective against the development of TB among patients who were newly diagnosed with type 2 DM; lower cumulative doses of metformin did not appear to reduce the incidence of active TB infection.

**Supplementary Information:**

The online version contains supplementary material available at 10.1186/s12890-021-01667-4.

## Background

Tuberculosis (TB) is a major global health problem. Active TB affects approximately 10 million individuals per year with a mortality rate of more than 1 million individuals per year [1]. In South Korea, 28,161 new TB cases were reported and the total number of notified patients with TB was 36,044 in 2017. Since 2010, TB public–private mix (PPM) program was implemented for more intensive TB control. This program includes free TB diagnosis, free treatment for TB, and allocation of TB-specialist nurses in all relevant public and private hospitals. Despite the effort, Korea still had high incidence rates of TB (77 per 100,000 people) as of 2016, which represents the highest incidence rates of TB among all member countries of the Organization for Economic Cooperation and Development (OECD) [2].

Approximately one in three individuals worldwide have a latent TB infection, most of whom never develop active TB during their lifetime. The lifetime risk of TB reactivation for a person with latent TB infection is 5–15%. Certain risk factors increase the probability that latent TB will progress to active TB; diabetes mellitus (DM) is one such risk factor [3, 4].

The association between DM and TB has been well documented. Patients with diabetes have a two- to three-fold higher risk of developing TB compared to individuals who have not been diagnosed with diabetes [5, 6]. Treatment failure and TB recurrence also are more frequent among patients with DM [5, 7–12]. Patients with DM have an impaired immune response, which facilitates both primary infection with *Mycobacterium tuberculosis* and reactivation of latent TB [13]. Diabetic hosts are slow to mount an innate response to the alveolar macrophages initially infected with *Mycobacterium tuberculosis*. This delay in innate immune response subsequently leads to downstream delays in adaptive immunity in the lung during the logarithmic growth phase of *M. tuberculosis* replication, which results in a higher plateau of lung bacterial load once effective control has been exerted. This higher plateau is associated with an increased severity of immune pathology and worse outcomes in patients with DM who develop TB [13, 14].

Metformin is generally prescribed as a first-line anti-diabetic agent due to its association with weight loss and its lack of association with hypoglycemic complications in patients with type 2 DM [15]. Beyond its hypoglycemic action, many experimental and clinical studies have reported on the pleiotropic effects of metformin, including in the prevention of atherosclerosis and the treatment of certain cancers and infections [16–19]. Metformin as a treatment for TB also has been actively studied. In particular, metformin is associated with the prevention of TB development, improvements in the successful treatment of TB, and decreases in the recurrence of TB among patients with DM [20–25]. These prior studies have some limitations, however, including small sample sizes, uncontrolled potential confounders, and cohorts that included both newly diagnosed and long-term patients with DM.

In this study, therefore, we evaluated the protective effect of metformin on the development of TB among newly diagnosed patients with type 2 DM. We used data from a large national database and controlled for several confounders.

## Methods

### Data source

This was a retrospective cohort study of the claims database of the Health Insurance Review and Assessment Service (HIRA, Seoul, South Korea), a government-affiliated agency that examines the accuracy of claims for the National Health Insurance (NHI, which covers ~ 97% of the South Korean population) and National Medical Aid (which covers ~ 3.5% of the South Korean population). The claims data of HIRA is collected when healthcare service providers submit a claim to HIRA to be reimbursed for a service that they provided to patients [26]. This HIRA database consists of 5 categories: (1) the general information (age, gender, insurance number, medical department visited, type of insurance, etc.); (2) treatment details (medical practice code, inpatient prescriptions, diagnostic test, operation, injection, etc.); (3) the diagnoses (all diagnoses); (4) the prescription (quantity per time, quantity per day, unit price, date of prescription, drug code, total days prescribed, drug generic name, manufacturer, channel of administration, etc.); (5) the provider information (provider ID, location, name of providers, types of provider, no. of beds, etc.) [27]. We used claims data that had been submitted by health care providers between 1 January 2002 and 31 December 2013. Anonymized identifiers were provided by the HIRA to protect privacy according to the Act on the Protection of Personal Information Maintained by Public Agencies.

### Study population

**Fig. 1:**
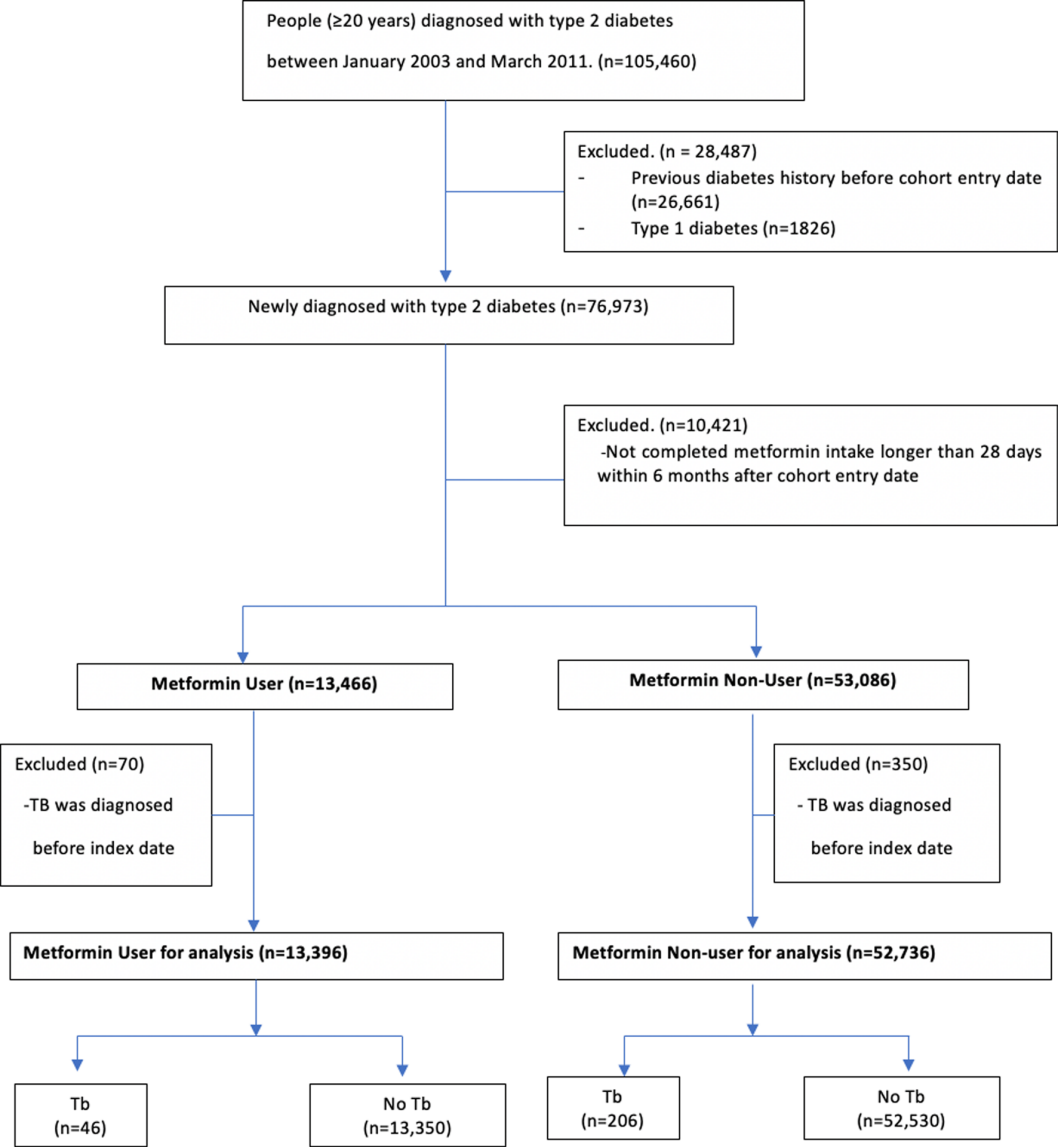
Flow chart

The study population included newly diagnosed patients with type 2 DM (ICD-10 codes E11-14) who were treated with anti-diabetic drugs between 1 January 2003 and 31 March 2011 and who were ≥ 20 years old at cohort entry (Fig. 1). We created and applied specific algorithms to identify the study populations more accurately from the claims database as below. Individuals with incident type 2 DM were defined according to the following eligibility criteria: (1) had at least two claims with ICD-10 code corresponding to type 2 DM (E11-E14) within one year or (2) at least one claim for a prescription for an anti-diabetic medication during the study period. Anti-diabetic drugs included biguanides (metformin), sulfonylureas, meglitinides, α-glucosidase inhibitors, insulin, thiazolidinediones, dipeptidyl peptidase-4 inhibitors, and glucagon-like peptide-1 (GLP-1) analogues (incretin). We excluded patients with type 1 DM, which was defined as those who had at least one claim with an ICD-10 E10 code and who were prescribed only insulin without any oral anti-diabetic drugs.

The principal exposure variable was metformin use. Metformin user is defined as a patient who had taken metformin for more than 28 days within the first 6 months after the initial cohort entry. A metformin non-user is defined as a patient who had never been treated with metformin during the study was conducted. Patients who had taken a metformin but did not meet the criteria of being a metformin user were excluded from the analysis. The index date for a metformin user was the 28th day after they started to take metformin within 6 months of entry. The index date for a metformin non-user was the 28th day after diagnosis with DM.

We subsequently excluded patients who had a TB before type 2 DM diagnosis. Exclusion criteria was defined as patients who had received a TB diagnosis and had taken anti-TB drugs based on ICD-10 codes for TB (A15–A19) within 1 year before the index date. Anti-TB drugs included isoniazid (INH), rifampicin (RMP), ethambutol (EMB), pyrazinamide (PZA), prothionamide (PTH), cycloserine (CS), para-aminosalicylic acid (PAS), Tubis® (INH, RMP, EMB and PZA combination drug), delamanid, and bedaquiline.

### Outcome variables

The main outcome variable was the incidence of TB among newly diagnosed patients with type 2 DM within 2 years after the index date. The diagnosis of TB was defined as both the use of ICD-10 codes for TB (A15–A19, U88.0–U88.1) and prescription of at least one of the following anti-TB drugs: (1) INH and RMP, (2) EMB, (3) PZA, (4) PTH, (5) CS, (6) PAS, (7) Tubis®, (8) delamanid, and (9) bedaquiline more than once within 90 days of the TB diagnosis. The event date was defined as the first day on which the anti-TB drug was prescribed.

### Data analysis

We compared the baseline characteristics of metformin users and non-users. The distributions of these characteristics were compared using Student’s *t* tests, Chi-square tests, or Fisher’s exact tests as appropriate. To account for significant differences in patient characteristics, we performed 1:1 propensity score (PS)—matched analysis. The PS was calculated by binary logistic models that included metformin use as the dependent variable and the following covariates as independent variables: age, sex, Charlson comorbidity index (CCI), healthcare utilization, anti-diabetic treatment, immunosuppressive treatment, and other comorbidities. Cox proportional hazard regression models were used to evaluate whether TB incidence differed between metformin users and PS-matched metformin non-users. We also investigated the relationship between metformin cumulative dose and TB incidence.

All drugs approved by the Ministry of Food and Drug Safety are assigned a 13-digit code in South Korea’s Health Insurance Review & Assessment Service (HIRA). This unified standard drug code system made it possible for us to calculate the total prescribed metformin dose during the study period. The cumulative metformin dose was calculated using the drug code containing the amount of metformin in each prescription and the total duration of metformin prescription.

Cumulative dose was categorized according to quartile (i.e., Q1, Q2, Q3, Q4). Characteristics that were significantly different between metformin users and non-users prior to the PS-matching were adjusted in the Cox proportional hazard regression models. We report hazard ratios (HRs) and 95% confidence intervals (CI) for metformin use both unadjusted and adjusted for age, sex, and other variables. All statistical analyses were performed using SAS version 9.4 (Statistical Analysis Software Institute; Cary, NC, USA).

### Ethical approval

This study involved the use of existing data coded in a manner that prevented the identification of patients either directly or through identifiers. The study protocol received a determination of exemption after review by the Institutional Review Board of Seoul National University Hospital (IRB No. 1906-048-1038).

## Results

In total, 76,973 patients were newly diagnosed with type 2 DM between January 1, 2003 and March 31, 2011. After excluding participants who did not meet the metformin-use definition or had a previous TB diagnosis, 66,132 patients were included in the analysis: 13,396 metformin users and 52,736 metformin non-users (Fig. 1). Baseline demographic and clinical characteristic data are shown in Table 1. Metformin users were younger and more male. Only 25% of metformin non-users were being treated with other anti-diabetic drugs, whereas the 89% of metformin users were concomitantly being treated with other anti-diabetic drugs. Comorbid risk factors for TB development, such as malignancy, malabsorption, chronic kidney disease, dialysis, gastrectomy, and organ transplantation, were more frequent among metformin non-users. Metformin non-users also were more likely to have chronic respiratory diseases and to be taking corticosteroids or other immunosuppressants. Metformin users had less frequent health care utilization compared to metformin non-users at baseline; this finding did not change significantly over the follow-up study period. Due to these differences in baseline characteristics between metformin users and non-users, propensity score (PS) matching was performed (Table 2) to generate two groups in which the standardized difference (STD) for any covariate was less than 10%.

**Table 1:** Baseline characteristics of patients

	Metformin user (n = 13,396)		Metformin non-user (n = 52,736)		*p* Value	STD
	Number	%	Number	%		
*Sex*						
Male	7681	57.34%	25,809	48.94%	< 0.001	16.89%
Female	5715	42.66%	26,927	51.06%		− 16.89%
*Age*^*1)*^						
20–29	216	1.61%	1375	2.61%	< 0.001	− 6.93%
30–39	1133	8.46%	4204	7.97%		1.77%
40–49	3064	22.87%	9797	18.58%		10.61%
50–59	3896	29.08%	13,410	25.43%		8.21%
60–69	3089	23.06%	13,024	24.70%		− 3.84%
70–79	1601	11.95%	8254	15.65%		− 10.74%
≥ 80	397	2.96%	2672	5.07%		− 10.73%
*Anti-diabetic treatment*^*2)*^						
Insulin therapy	1585	11.83%	2228	4.22%	< 0.001	28.27%
Sulfonylurea	8253	61.61%	8260	15.66%	< 0.001	107.02%
Other drugs(excluding Metformin)	4630	34.56%	2685	5.09%	< 0.001	79.55%
*Anti-diabetic treatment*^*3)*^						
Insulin therapy	1439	10.74%	3828	7.26%	< 0.01	12.19%
Sulfonylurea	7811	58.31%	8071	15.30%	< 0.01	99.62%
Other drugs(exclusingMetformin)	2651	19.79%	2013	3.82%	< 0.01	51.09%
*Other comorbidities*^*2)*^						
Malignancy	581	4.34%	4429	8.40%	< 0.001	− 16.69%
Malabsorption	11	0.08%	118	0.22%	0.001	− 3.63%
Chronic kidney disease	32	0.24%	871	1.65%	< 0.001	− 14.64%
Dialysis	3	0.02%	290	0.55%	< 0.001	− 9.89%
Gastrectomy	5	0.04%	111	0.21%	< 0.001	− 4.92%
HIV/AIDS & organ transplantation	11	0.08%	126	0.24%	< 0.001	− 3.9%
*Immunosuppresives*^*2)*^						
Systemic corticosteroids	4796	35.80%	24,924	47.26%	< 0.001	− 23.42%
Other immunosuppressants	280	2.09%	2015	3.82%	< 0.001	− 10.23%
*Immunosuppresives*^*3)*^						
Systemic corticosteroids	6605	49.31%	31,187	59.14%	< 0.001	− 19.83%
Other immunosuppressants	475	3.55%	2839	5.38%	< 0.001	− 8.91%
*Charlson comorbidity index*^*2)*^						
Mean ± SD	2.36	1.71	2.56	1.96	< 0.001	
Median(Q1, Q3)	2	(1, 3)	2	(1, 3)		
0–1	4645	34.67%	17,367	32.93%	< 0.001	3.68%
2–3	5926	44.24%	22,428	42.53%		3.45%
≥ 4	2825	21.09%	12,941	24.54%		− 8.23%
*Healthcare utilization*^*2)*^						
Hospitalization, days						
Mean ± SD	0.340	0.949	0.495	1.263		
Median(Q1, Q3)	0	(0, 0)	0	(0, 1)		
0	10,593	79.08%	39,000	73.95%	< 0.001	12.11%
1	1905	14.22%	8160	15.47%		− 3.52%
2–3	733	5.47%	4296	8.15%		− 10.63%
≥ 4	165	1.23%	1280	2.43%		− 8.93%
*Outpatient visit, days*						
Mean ± SD	17.448	20.793	22.670	24.343	< 0.001	
Median(Q1, Q3)	11	(5, 22)	16	(8, 29)		
< 15	8327	62.16%	25,994	49.29%	< 0.001	26.13%
16–30	3065	22.88%	14,562	27.61%		− 10.91%
31–50	1249	9.32%	7296	13.83%		− 14.13%
> 50	755	5.64%	4884	9.26%		− 13.84%
*Healthcare utilization*^*3)*^						
Hospitalization, days						
Mean ± SD	0.714	2.243	0.954	2.766		
Median(Q1, Q3)	0	(0, 1)	0	(0, 1)		
0	9718	72.54%	35,745	67.78%	< 0.001	10.42%
1	1967	14.68%	8568	16.25%		− 4.33%
2–3	1121	8.37%	5017	9.51%		− 4.01%
≥ 4	590	4.40%	3406	6.46%		− 9.07%
Outpatient visit, days						
Mean ± SD	43.556	43.298	47.169	50.329	< 0.001	
Median(Q1, Q3)	33	(19, 54)	34	(17, 60)		
< 15	2620	19.56%	12,103	22.95%	< 0.001	− 8.30%
16–30	3492	26.07%	11,740	22.26%		8.90%
31–50	3550	26.50%	11,836	22.44%		9.45%
> 50	3734	27.87%	17,057	32.34%		− 9.76%

^1)^Age at cohort entry date

^2)^Within 1-year prior to index date

^3)^Within follow-up period (During 2-year from index date or until TB development)

**Table 2:** Comparison of baseline characteristics in 1:1 matched cohort between metformin user and non-user

	Metformin user (n = 12,916)		Metformin non-user (n = 12,916)		*p* Value	STD
	Number	%	Number	%		
*Sex*						
Male	7377	57.12	7378	57.12	0.990	− 0.02%
Female	5539	42.88	5538	42.88		0.02%
*Age*^*1)*^						
20–29	210	1.63	174	1.35	< 0.001	2.30%
30–39	1086	8.41	913	7.07		5.01%
40–49	2932	22.70	2694	20.86		4.47%
50–59	3750	29.03	3611	27.96		2.38%
60–69	2986	23.12	3156	24.43		− 3.09%
70–79	1559	12.07	1854	14.35		− 6.75%
≥ 80	393	3.04	514	3.98		− 5.09%
*Anti-diabetic treatment*^*2)*^						
Insulin therapy	1319	10.21	1078	8.35	< 0.001	6.43%
Sulfonylureas	7331	56.76	7650	59.23	< 0.001	− 5.01%
Other drugs (excluding metformin)	2190	16.96	1797	13.91	< 0.001	8.43%
*Other comorbidities*^*2)*^						
Malignancy	565	4.37	615	4.76	0.136	− 1.85%
Malabsorption	11	0.09	7	0.05	0.346	1.17%
Chronic kidney disease	32	0.25	36	0.28	0.627	− 0.60%
Dialysis	3	0.02	1	0.01	0.625	1.24%
Gastrectomy	4	0.04	5	0.03	1.000	− 0.41%
HIV and Organ transplantation	14	0.11	11	0.09	0.548	0.75%
*Immunosuppresives*^*2)*^						
Systemic corticosteroids	4659	36.07	4690	36.31	0.688	− 0.5%
TNF alpha + Other immunosuppressant	275	2.13	268	2.07	0.761	0.38%
*Charlson comorbidity index*^*2)*^						
Mean ± SD	2.34	1.70	2.24	1.75	< 0.001	
Median (Q1,Q3)	2	(1,3)	2	(1,3)		
0–1	4511	34.93	4903	37.96	< 0.001	− 6.31%
2–3	5719	44.28	5489	42.50		3.59%
≥ 4	2686	20.80	2524	19.54		3.13%
*Healthcare utilization*^*2)*^						
Hospitalization, days						
Mean ± SD	0.34	0.95	0.32	0.91	0.169	
Median (Q1,Q3)	0	(0,0)	0	(0,0)		
0	10,237	79.26	10,327	79.96	0.458	− 1.73%
1	1818	14.08	1780	13.78		0.85%
2–3	701	5.43	652	5.05		1.70%
≥ 4	160	1.24	157	1.22		0.21%
Outpatient visit, days						
Mean ± SD	17.59	20.93	16.98	20.17	0.018	
Median (Q1,Q3)	11	(5,22)	11	(5,22)		
0	7986	61.83	8164	63.21	0.147	− 2.85%
1	2968	22.98	2858	22.13		2.04%
2–3	1223	9.47	1191	9.22		0.85%
≥ 4	739	5.72	703	5.44		1.21%

*STD* standardized difference

^1)^Age at cohort entry

^2)^Within 1-year prior to index date

^3)^Within follow-up period (During 2-year from index date or until TB development)

**Fig. 2:**
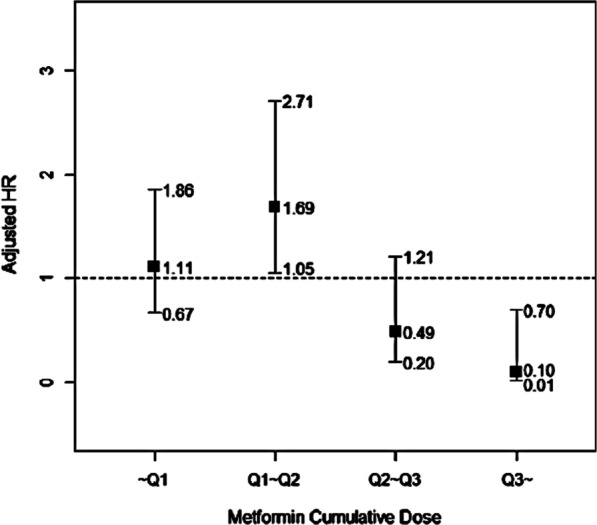
Adjusted hazard ratio (HR) of TB development according to cumulative dose of metformin. *Adjusted for age, sex, the use of insulin, sulfonylurea, other anti-diabetic treatment excluding metformin, systemic corticosteroid, other immunosuppressants, and comorbidities including malignancy, malabsorption, CKD, dialysis, gastrectomy, HIV/AIDS and organ transplantation, CCI, number of hospitalization, and outpatient visit days. HR = hazard ratio; CI = confidence interval; CKD = chronic kidney disease; CCI = charlson comorbidity index

PS-matched Cox proportional hazard regression models showed that metformin use was not associated with the prevention of TB development among patients who were newly diagnosed with type 2 DM (HR 1.17; 95% CI 0.75–1.83; *P* = 0.482) (Table 3). A trend towards the prevention of TB development was observed for higher cumulative doses of metformin at the two highest quartiles (Q3 and Q4) compared to metformin non-users (*P* value for trend = 0.059) (Fig. 2). The risk of TB development was only 10% among patients in the highest quartile (Q4) compared to metformin non-users. In contrast, however, the risk of TB was higher in patients in the 2nd quartile (Q2) compared to patients in the 1st quartile (Q1).

**Table 3:** Association between metformin use and the risk of TB development in 1:1 matched cohort

	Tb development			Univariate			Adjusted*		
	n	N	%	HR	95% CI	*p* Value	HR	95% CI	*p* Value
*Any Metformin (*≥ *28d within 6mo)*									
Non-user	40	12,916	0.31	Ref			Ref		
User	44	12,916	0.34	1.10	0.72–1.69	0.663	1.17	0.75–1.83	0.482
*Age*^*1)*^									
20–29	1	384	0.26	1			1		
30–39	6	1999	0.30	1.15	0.14–9.59	0.894	0.81	0.26–2.55	0.722
40–49	11	5626	0.20	0.75	0.10–5.82	0.785	0.79	0.28–2.29	0.669
50–59	23	761	0.31	1.20	0.16–8.90	0.857	1.46	0.53–4.06	0.466
60–69	17	6142	0.28	1.06	0.14–8.00	0.952	1.77	0.64–4.92	0.271
70–79	19	3413	0.56	2.14	0.29–16.01	0.458	2.22	0.80–6.18	0.126
≥ 80	8	907	0.88	3.40	0.43–27.21	0.248	2.47	0.86–7.09	0.093
*Sex*^*1)*^									
Male	57	14,755	0.39	1			1		
Female	27	11,077	0.24	0.63	0.40–1.00	0.048	0.77	0.59–1.00	0.053
*Comorbidities*^*3)*^									
Malignancy	11	1798	0.61	2.02	1.07–3.80	0.030	1.24	0.61–2.52	0.556
HIV/AIDS & organ transplantation	1	37	2.70	8.60	1.20–61.77	0.032	9.36	1.16–75.72	0.036
Malabsorption	0	51	0.00	0	0	0.978	0	0	0.987
Chronic kidney disease	2	243	0.82	2.57	0.63–10.44	0.188	1.53	0.37–6.35	0.562
Gastrectomy	0	20	0.00	0	0	0.983	0	0	0.995
*Immunosuppresives*^*3)*^									
Systemic corticosteroids	42	12,929	0.32	1.0	0.65–1.53	0.990	1.31	0.81–2.10	0.272
Other immunosuppressants	4	938	0.43	1.33	0.49–3.62	0.580	0.7	0.25–2.00	0.508
*Charlson comorbidity index*^*3)*^									
0–1	26	9414	0.28	1			1		
2–3	32	11,208	0.29	1.03	0.62–1.74	0.900	1.48	1.03–2.12	0.032
≥ 4	26	5,210	0.50	1.81	1.05–3.12	0.033	1.60	1.07–2.39	0.023
*Anti-diabetic treatment*^*3)*^									
Insulin therapy	24	2,384	1.01	3.95	2.46–6.34	< 0.001	1.69	0.97–2.94	0.06
Sulfonylurea	48	13,924	0.34	1.14	0.74–1.76	0.552	1.19	0.75–1.87	0.46
Other drugs	25	6,224	0.40	1.34	0.84–2.13	0.225	1.27	0.77–2.08	0.34
*Healthcare utilization*^*3)*^									
Hospitalization, days									
0	32	18,900	0.17	1			1	1	
1	26	3,713	0.70	4.15	2.47–6.96	< 0.001	4.59	2.63–7.99	< 0.001
2–3	18	2,069	0.87	5.16	2.89–9.18	< 0.001	5.51	2.85–10.67	< 0.001
≥ 4	8	1,150	0.70	4.12	1.90–8.93	< 0.001	3.18	1.34–7.56	0.001
Outpatient visit, days									
< 15	41	6,142	0.67	1			1	1	
16–30	17	6,551	0.26	0.39	0.22–0.68	< 0.001	0.33	0.18–0.58	< 0.001
31–50	10	6,290	0.16	0.24	0.12–0.47	< 0.001	0.16	0.08–0.32	< 0.001
> 50	16	6,849	0.23	0.35	0.20–0.62	< 0.001	0.17	0.09–0.32	< 0.001

*Adjusted for all variables in the table

^1)^Age at cohort entry date

^2)^Within 1-year prior to index date

^3)^Within follow-up period (During 2-year from index date or until TB development)

## Discussion

In our study, metformin use was not associated with reducing the risk of incident TB among patients who were newly diagnosed with type 2 DM. Our data suggest, however, that a higher cumulative dose of metformin may protect against the development of TB. Any metformin use was not significantly associated with the reducing the risk of incident TB in multivariate analysis (adjusted HR 0.93; 95% CI 0.65–1.34) (Additional file 1: Supplementary Table 1) and in PS-matched participants (HR 1.17; 95% CI 0.75–1.83) across the total study period (Table 3). Intriguingly, however, we observed two phases of metformin cumulative dose that were associated with the development of TB. Among patients in the 2nd quartile (Q2) of metformin cumulative dose, the HR for TB development was 1.69 (95% CI, 1.05–2.71; *P* = 0.030) (Fig. 2).In contrast, the HR for the development of TB trended towards a reduction among patients in the 3rd quartile (Q3) of metformin cumulative dose (HR 0.49; 95% CI 0.20–1.21, *P* = 0.030) and a significant reduction among patients in the 4th quartile (Q4) of metformin cumulative dose (HR 0.10; 95% CI 0.01–0.70, *P* = 0.021). The effects at these two different time periods may have combined to produce null findings in the overall model.

These findings may reveal different effects of metformin on the development of TB. During the early phase of metformin treatment, metformin may disturb anti-TB immunity. According to one report, metformin downregulated TNF-α production and excretion, which is an important cytokine for both macrophage activation and granuloma formation in obese mice [28] and macrophages [29]. Similarly, a study in a mouse model of TB reported that bacillary load increased within the first 2 weeks of metformin treatment, followed by an anti-TB effect thereafter [30]. In another experimental model, metformin did not initially improve the sterilizing activity of a first-line anti-TB treatment in mice; however, after 3.5 months of treatment, the addition of metformin to standard therapy reduced mean lung bacillary load by 0.18 log_10_ compared to a group receiving standard therapy only (*P* = 0.039) [31]. In human in vitro and in vivo studies, metformin inhibited a type I interferon (IFN) response induced by *M*. *tuberculosis*, and both IFN-ϒ and TNF-α were reduced for up to 21 days after metformin intake. In this same study, however, metformin increased phagocytic activity and reactive oxygen species production [32]. Based on these results, there are two possible roles of metformin during the early phases of treatment: (1) metformin may disturb anti-TB immunity by down-regulating IFN-ϒ and TNF-α; and (2) metformin may suppress the sterilizing effects of anti-TB agents during early metformin treatment. In contrast, during later phase of treatment, metformin may restrict mycobacterial growth by inducing mitochondrial reactive oxygen species production and phagocytic activity [30].

According to the study from Taiwan, metformin use was associated with a reduced risk of TB infection in type 2 DM patients (overall HR 0.552; 95% CI 0.493–0.617). Metformin showed an anti-TB effect in dose–response manner. However, this anti-TB effect of metformin was not significant in the first tertile group (< 27.10 months) of cumulative duration and the first tertile group (< 817,000 mg) of cumulative dose of metformin [HR 1.116 (0.989–1.261), 1.037 (0.918–1.173), respectively] [33]. This study also suggests the early phase of metformin therapy may disturb anti-TB immunity.

Lin et al. [21] reported that metformin use independently reduced the risk of the development of TB (RR 0.24; 95% CI 0.11–1.87), which is not consistent with our results. This study analyzed 5026 PS-matched metformin users and non-users among newly diagnosed patients with type 2 DM from the Taiwan claims database between 1998 and 2010. In contrast to our study, however, the clinical characteristics between metformin users and non-users remained unbalanced even after PS-matching. For example, 56.9% of metformin users had been treated with statins, which may independently protect against the development of TB [34]. Moreover, the time since DM diagnosis was not well controlled in their study population; the risk of developing TB was more than two-fold higher among patients who had DM for over six years compared to patients who had DM for less than six years. Our study controlled for these potential confounders by balancing statin treatment between metformin users and non-users and only enrolling patients who were newly diagnosed with DM.

Despite the results reported above, a different retrospective study using claims data from Taiwan showed that metformin use was an independent factor for preventing the development of TB compared to sulfonylurea use (HR 0.337; 95% CI 0.169–0.673) [23].This study did not compare metformin users to non-users, however. This study showed there were more rural residents with less statin users in the sulfonylurea group, which is associated with the development of TB. However, even after PS matching was conducted to control for the differences, they remained statistically significant between the two groups. In addition, it has been reported that sulfonylurea may increase the risk of infection [35]. Sulfonylurea reduced primary human monocyte functions in response to TB in an in vitro study of patients with type 2 DM. Treatment with sulfonylurea therefore may result in an increased susceptibility to TB among patients with type 2 DM [36]. Our study controlled for these confounders by including other drugs, including anti-diabetic drugs and immunosuppressives, in PS matching between metformin users and non-users.

However, our claims database did not have laboratory records and we did not know the actual severity of hyperglycemia. Another limitation is there may be a discrepancy between the prescription quantity of the drug and the actual drug quantity of administered by patients. And discrepancies could occur between diagnosis entered in the data and actual diseases that a patient had. For example, some physician dose not submit TB diagnosis and dose not start anti-TB treatment until he obtains a microbiological result. Other physician may start anti-TB medication empirically in DM patients based only on typical chest imaging of TB.

Therefore, further randomized controlled study is needed to find out the effect of metformin on the risk of incident TB in type 2 DM patients.

## Conclusion

Metformin use was not associated with reducing the risk of TB in patients who were newly diagnosed with type 2 DM in our study. Among patients with lower cumulative doses, metformin trended towards an increased risk of TB, whereas higher cumulative doses decreased the risk of TB. These findings suggest that higher doses and longer durations of metformin may lower the risk of incident TB in patients with type 2 DM.

## Supplementary Information

**Additional file 1. Supplementary Table 1.** The effecf ot metformin use on the development of TB

## Data Availability

The datasets generated and analyzed during the current study are not publicly available due [REASON WHY DATA ARE NOT PUBLIC] but are available from the corresponding author on reasonable request.

## Abbreviations

TB: Tuberculosis
DM: Diabetes mellitus
PS: Propensity score
HIRA: The Health Insurance Review and Assessment Service
NHI: The National Health Insurance
ICD: International Classification of Disease
GLP-1: Glucargon-like peptide-1
INH: Isoniazid
RMP: Rifampicin
EMB: Ethambutol
PZA: Pyrazinamide
PTH: Prothionamide
CS: Cycloserine
PAS: Para-aminosalicylic acid
CCI: Charlson comorbidity index
HR: Hazard ratios
CI: Confidence intervals
STD: Standardized difference
