# Supplementary material for: The cumulative dose-dependent effects of metformin on the development of tuberculosis in patients newly diagnosed with type 2 diabetes mellitus
Source: BMC Pulm Med. 2021 Sep 25;21:303. doi: 10.1186/s12890-021-01667-4 (PMC8464151; doi:10.1186/s12890-021-01667-4)
Supplement: Supplementary file 1 — Additional file 1. Supplementary Table 1. The effecf ot metformin use on the development of TB [file 12890_2021_1667_MOESM1_ESM.docx]

**Supplementary Table 1. The effect of metformin use on the development of TB**

|  | | **Univariate** | | | **Adjusted** | | |
| --- | --- | --- | --- | --- | --- | --- | --- |
|  |  | **HR** | **95% CI** | **P-value** | **HR** | **95% CI** | **P-value** |
| **Metformin use** | | 0.88 | 0.64,1.21 | 0.428 | 0.93 | 0.65,1.34 | 0.694 |
| **Age^1)^** | 20-29 | 1 |  |  | 1 |  |  |
|  | 30-39 | 0.82 | 0.26,2.57 | 0.733 | 0.81 | 0.26,2.55 | 0.722 |
|  | 40-49 | 0.77 | 0.27,2.22 | 0.632 | 0.79 | 0.28,2.29 | 0.669 |
|  | 50-59 | 1.29 | 0.47,3.55 | 0.626 | 1.46 | 0.53,4.06 | 0.466 |
|  | 60-69 | 1.58 | 0.58,4.34 | 0.374 | 1.77 | 0.64,4.92 | 0.271 |
|  | 70-79 | 2.47 | 0.90,6.78 | 0.080 | 2.22 | 0.80,6.18 | 0.126 |
|  | ≥ 80 | 4.04 | 1.42,11.43 | 0.009 | 2.47 | 0.86,7.09 | 0.093 |
| **Sex** | Male | 1 |  |  | 1 |  |  |
|  | Female | 0.70 | 0.54,0.90 | 0.005 | 0.77 | 0.59,1.00 | 0.053 |
| **Anti-diabetic treatment ^3)^** | insulin | 3.28 | 2.35,4.57 | <0.001 | 1.13 | 0.78,1.65 | 0.514 |
|  | sulfonylurea | 1.07 | 0.81,1.41 | 0.655 | 1.10 | 0.79,1.52 | 0.587 |
|  | others | 1.13 | 0.78,1.64 | 0.529 | 1.12 | 0.73,1.71 | 0.611 |
| **Number of hospitalization^3)^** | 0 | 1 |  |  | 1 |  |  |
|  | 1 | 4.27 | 3.13,5.83 | <0.001 | 4.35 | 3.12,6.07 | <0.001 |
|  | 2-3 | 6.04 | 4.36,8.38 | <0.001 | 5.59 | 3.83,8.16 | <0.001 |
|  | ≥4 | 3.99 | 2.59,6.13 | <0.001 | 2.55 | 1.56,4.18 | <0.001 |
| **Outpatient visit, days^3)^** | <15 | 1 |  |  | 1 |  |  |
|  | 16-30 | 0.50 | 0.36,0.69 | <0.001 | 0.48 | 0.35,0.67 | <0.001 |
|  | 31-50 | 0.36 | 0.26,0.52 | <0.001 | 0.28 | 0.19,0.41 | <0.001 |
|  | >50 | 0.28 | 0.19,0.39 | <0.001 | 0.14 | 0.10,0.21 | <0.001 |
| **Charlson comorbidity index^2)^** | 0-1 | 1 |  |  | 1 |  |  |
|  | 2-3 | 1.50 | 1.06,2.02 | 0.021 | 1.48 | 1.03,2.12 | 0.032 |
|  | ≥4 | 2.64 | 1.91,3.66 | <0.001 | 1.60 | 1.07,2.39 | 0.023 |
| **Immunos-uppressives^3)^** | Systemic corticosteroid | 0.82 | 0.64,1.06 | 0.124 | 1.06 | 0.81,1.39 | 0.679 |
|  | Other immunosuppressives | 1.55 | 0.97,2.47 | 0.068 | 0.83 | 0.49,1.40 | 0.476 |
| **Other comorbidities^3)^** | Malignancy | 2.44 | 1.79,3.31 | <0.001 | 1.24 | 0.85,1.79 | 0.261 |
|  | HIV/AIDS & organ transplantation | 9.37 | 1.32,66.74 | 0.026 | 5.45 | 0.75,39.48 | 0.093 |
|  | Malabsoprtion | 1.26 | 0.18,8.98 | 0.818 | 0.95 | 0.13,6.85 | 0.957 |
|  | CKD | 3.46 | 2.05,5.83 | <0.001 | 1.41 | 0.71,2.80 | 0.324 |
|  | Dialysis | 6.87 | 3.53,13.35 | <0.001 | 2.96 | 1.24,7.03 | 0.014 |
|  | Gastrectomy | 2.27 | 0.32,16.15 | 0.413 | 1.08 | 0.15,7.91 | 0.939 |

| 1) Age at cohort entry date |  |  |  |  |
| --- | --- | --- | --- | --- |
| 2) Within 1-year prior to index date | | | | |
| 3) Within follow-up period (During 2-year from index date or until TB development) | | | | |
